# Cyclosporine A and tacrolimus inhibit bladder cancer growth through down-regulation of NFATc1

**DOI:** 10.18632/oncotarget.2750

**Published:** 2015-01-08

**Authors:** Takashi Kawahara, Eiji Kashiwagi, Hiroki Ide, Yi Li, Yichun Zheng, Yurina Miyamoto, George J. Netto, Hitoshi Ishiguro, Hiroshi Miyamoto

**Affiliations:** ^1^ Departments of Pathology and Urology, Johns Hopkins University School of Medicine, Baltimore, MD, USA; ^2^ Department of Pathology and Laboratory Medicine, University of Rochester Medical Center, Rochester, NY, USA

**Keywords:** bladder cancer, cyclosporine, NFAT, tacrolimus, tumor progression

## Abstract

The functional role of nuclear factor of activated T-cells (NFAT), a key regulator of the immune response, in bladder cancer progression remains uncertain. In this study, we assessed biological significance of NFAT in human bladder cancer. Immunohistochemistry detected nuclear/cytoplasmic NFATc1 signals in 14 (21.5%)/34 (52.3%), respectively, of 65 muscle-invasive bladder carcinomas and showed that patients with nuclear NFATc1-positive tumor had a significantly higher risk of disease progression (*P* = 0.006). In bladder cancer cell lines, cyclosporine A (CsA) and tacrolimus (FK506), immunosuppressant drugs/non-selective NFAT inhibitors, attenuated NFATc1 expression and its nuclear translocation, NFAT transcriptional activity, and the expression of cyclooxygenase-2 and c-myc, downstream targets of NFATc1. NFAT inhibition via NFATc1-small interfering RNA (siRNA) or treatment with these NFAT inhibitors resulted in decreases in cell viability/colony formation, cell migration/invasion, and the expression/activity of MMP-2 and MMP-9, as well as an increase in apoptosis, in the parental/control lines. No significant additional inhibition in the viability and invasion of NFATc1-siRNA cells was seen. In xenograft-bearing mice, CsA and FK506 significantly retarded tumor growth. These results suggest that NFATc1 plays an important role in bladder cancer outgrowth. Thus, NFATc1 inactivation, especially using CsA and FK506, has the potential of being a therapeutic approach for bladder cancer.

## INTRODUCTION

Nuclear factor of activated T-cells (NFAT), initially identified as a regulator of T-cell activation [1], has subsequently been characterized mainly in immune cells [2]. NFAT family members, including NFATc1 (also known as NFAT2), NFATc2 (NFAT1), NFATc3 (NFAT4), and NFATc4 (NFAT3), are located in the cytoplasm of immune cells (*e.g*. lymphocyte) in a hyperphosphorylated state and translocate into the nucleus upon cell stimulation by their dephosphorylation in response to Ca^2+^-calcineurin signals [2, 3]. The nuclear NFATs then form heterodimers with other transcription factors and induce downstream gene transcription. Interest in NFAT signaling was further enhanced by the observation indicating that cyclosporine A (CsA) and tacrolimus (FK506), immunosuppressants widely used in transplant medicine, specifically inactivated the NFAT pathway [4].

There is an increasing amount of evidence suggesting that NFAT signaling contributes to the development and progression of not only hematological malignancies but also solid tumors. Expression levels of NFAT isoforms, especially NFATc1, have been assessed in several types of cancer tissue specimens. Indeed, NFATc1 overexpression was detected in pancreatic [5], lung [6], and liver [7] carcinomas. Further functional analyses have revealed NFAT isoform-specific involvement in cancer progression, as seen in the regulation of hematopoietic system. In particular, NFATc1 was shown to promote tumor growth via, for instance, induction of c-myc in pancreatic cancer [5], cyclooxygenase (COX)-2 in melanoma [8], and autotaxin/lysophosphatidic acid axis in breast cancer [9]. In a sarcoma model, NFATc1 was also found to be oncogenic, whereas NFATc2 functioned as a tumor suppressor [10]. Moreover, NFATc1 has been implicated, as a key regulator, in lymphangiogenesis [11]. On the other hand, little is known about the specific roles of NFATc3, NFATc4, and NFAT5 in tumorigenesis and tumor progression, although inhibition of breast cancer cell motility by NFATc4 has been reported [12].

The functional role of NFAT signaling in bladder cancer progression remains largely unknown. In the current study, we investigated whether NFATc1 inactivation inhibited the growth of bladder cancer cells.

## RESULTS

### Expression of NFATc1 in human bladder cancer

**Figure 1:**
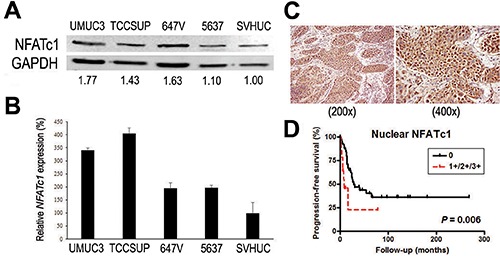
Expression of NFATc1 in bladder cancer **(A)** Western blotting of NFATc1 in urothelial cell lines. Total protein extracted from each cell line was immunoblotted for NFATc1 (105 kDa). GAPDH (37 kDa) served as an internal control. Densitometry values for specific bands standardized by GAPDH that are relative to the value in SVHUC cells are included below the lanes. **(B)** Quantitative RT-PCR of *NFATc1* in urothelial cell lines. Total RNA isolated from each cell line was subjected to real-time RT-PCR. Expression of *NFATc1* gene was normalized to that of GAPDH. Transcription amount is presented relative to that in SVHUC. Each value represents the mean (+SD) from at least three independent experiments. **(C)** Immunohistochemistry of NFATc1 in bladder cancer specimens. **(D)** Progression-free survival rates in patients with invasive bladder cancer. Kaplan-Meier analysis was performed according to the expression of NFATc1, and comparison was made by log-rank test.

We first examined the expression of NFATc1 in human urothelial carcinoma cell lines, UMUC3, TCCSUP, 647V, and 5637, and an immortalized human normal urothelial cell line, SVHUC, by western blotting (Fig. 1A) and reverse transcription (RT)-polymerase chain reaction (PCR) (Fig. 1B). All four bladder cancer lines were found to express NFATc1 at both protein and mRNA levels, and its expression in the benign urothelial line was weaker.

We next stained immunohistochemically for NFATc1 in the bladder tissue microarrays (TMAs) consisting of 65 cases of invasive bladder cancer. Positive signals were detected in both the nucleus and cytoplasm of tumor cells (Fig. 1C). Nuclear NFATc1 was positive in 14 cases [21.5%; 9 (13.8%) 1+, 4 (6.2%) 2+, and 1 (1.5%) 3+] and cytoplasmic NFATc1 was positive in 34 cases [52.3%; 19 (29.2%) 1+, 8 (12.3%) 2+, and 7 (10.8%) 3+] (Table 1). Spearman's correlation analysis revealed a positive correlation between nuclear versus cytoplasmic expression of NFATc1 (R^2^ = 0.2117; *P* < 0.001). Meanwhile, there were no statistically significant correlations between nuclear or cytoplasmic positivity of NFATc1 and patient age, gender, pT stage, or status of lymph node involvement. Kaplan-Meier analysis coupled with log-rank test further showed a strong association between nuclear expression of NFATc1 (*P* = 0.006; Fig. 1D), but not its cytoplasmic positivity (*P* = 0.164; figure not shown), and disease progression after cystectomy.

**Table 1:** Expression of NFATc1 in bladder cancer tissue microarrays

	*n*	Expression levels				*P* value
		Negative		Positive		
		0	1+	2+	3+	0 *vs*. 1+/2+/3+
Nucleus						
Total	65	51 (78.5%)	9 (13.8%)	4 (6.2%)	1 (1.5%)	
pT2	23	18 (78.3%)	4 (17.4%)	0 (0.0%)	1 (4.3%)	0.920
pT3	31	25 (80.6%)	4 (12.9%)	2 (6.5%)	0 (0.0%)	
pT4	11	8 (72.7%)	1 (9.1%)	2 (18.2%)	0 (0.0%)	
pN0	39	30 (76.9%)	7 (17.9%)	1 (2.6%)	1 (2.6%)	1.000
pN+	21	16 (76.2%)	2 (9.5%)	3 (14.3%)	0 (0.0%)	
Cytoplasm						
Total	65	31 (47.7%)	19 (29.2%)	8 (12.3%)	7 (10.8%)	
pT2	23	12 (52.2%)	7 (30.4%)	2 (8.7%)	2 (8.7%)	0.331
pT3	31	16 (51.6%)	8 (25.8%)	3 (9.7%)	4 (12.9%)	
pT4	11	3 (27.3%)	4 (36.4%)	3 (27.3%)	1 (9.1%)	
pN0	39	22 (56.4%)	11 (28.2%)	4 (10.3%)	2 (5.1%)	0.109
pN+	21	7 (33.3%)	7 (33.3%)	2 (9.5%)	5 (23.8%)	

### Down-regulation of NFAT by CsA and FK506 in bladder cancer cells

**Figure 2:**
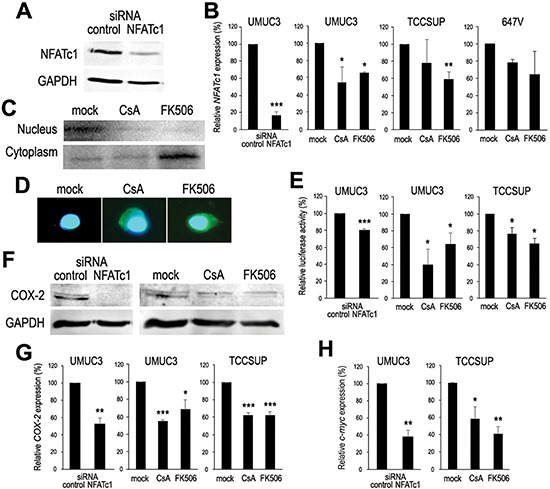
Inactivation of NFATc1 in bladder cancer **(A)** Western blotting of NFATc1 in UMUC3 cells transfected with control-siRNA or NFATc1-siRNA. Cell extracts were immunoblotted for NFATc1 (105 kDa). GAPDH (37 kDa) served as an internal control. **(B)** Quantitative RT-PCR of *NFATc1* in bladder cancer cells. UMUC3 cells expressing control-siRNA or NFATc1-siRNA and UMUC3/TCCSUP/647V cells treated with ethanol (mock), CsA (1 μM), or FK506 (1 μM) for 24 hours were subjected to RNA extraction and subsequent real-time RT-PCR. Expression of *NFATc1* gene was normalized to that of GAPDH. Transcription amount is presented relative to that of control-siRNA expression or mock treatment in each cell line. Each value represents the mean (+SD) from at least three independent experiments. *P < 0.05 (*vs*. mock treatment). **P < 0.01 (*vs*. mock treatment). ***P < 0.001 (*vs*. control-siRNA). **(C)** Western blotting of NFATc1 in UMUC3 cells treated by ethanol (mock), CsA (1 μM), or FK506 (1 μM) for 24 hours. Separate nuclear and cytoplasmic protein fractions were immunoblotted for NFATc1. **(D)** Immunofluorescent staining of NFATc1 in UMUC3 cells treated with ethanol (mock), CsA (1 μM), or FK506 (1 μM), along with ionomycin (1 μM) to induce nuclear translocation of NFATc1, for 30 minutes before formalin fixation. We merged the images between NFATc1 and DAPI that was used to visualize nuclei. Cytoplasmic signals of NFATc1 are seen in the CsA- and FK506-treated cells, but not in the mock-treated cell. **(E)** NFAT luciferase reporter activity in bladder cancer cells. UMUC3, with or without co-expression of control-siRNA or NFATc1-siRNA, or TCCSUP cells were transfected with pGL4.30-NFAT-response element and pRL-TK and subsequently cultured with ethanol (mock), CsA (1 μM), or FK506 (1 μM) for 24 hours. Luciferase activity is presented relative to that of control-siRNA expression or mock treatment in each cell line. Each value represents the mean (+SD) from at least three independent experiments. *P < 0.05 (*vs*. mock treatment in each cell line). ***P < 0.001 (*vs*. control-siRNA). **(F)** Western blotting of COX-2 in UMUC3 cells either transfected with control-siRNA or NFATc1-siRNA or treated with ethanol (mock), CsA (1 μM), or FK506 (1 μM) for 24 hours. Cell extracts were immunoblotted for COX-2 (69 kDa) and GAPDH. Quantitative RT-PCR of *COX-2* **(G)** and *c-myc* **(H)** in bladder cancer cells. UMUC3 cells expressing control-siRNA or NFATc1-siRNA and UMUC3/TCCSUP cells treated with ethanol (mock), CsA (1 μM), or FK506 (1 μM) for 24 hours were subjected to RNA extraction and subsequent real-time RT-PCR. Expression of  *COX-2* or *c-myc* gene was normalized to that of GAPDH. Transcription amount is presented relative to that of control-siRNA expression or mock treatment in each cell line. Each value represents the mean (+SD) from at least three independent experiments. *P < 0.05 (*vs*. mock treatment in each cell line). **P < 0.01 (*vs*. control-siRNA or mock treatment in each cell line). ***P < 0.001 (*vs*. mock treatment in each cell line).

We determined the effects of CsA and FK506, which were known to inhibit NFAT signals in immune cells [4], on NFATc1 expression by western blotting, RT-PCR, and immunofluorescence in bladder cancer cells. NFAT-mediated transcriptional activity was also determined in the cell extracts with transfection of a NFAT luciferase reporter plasmid. As expected, transfection of a NFATc1-small interfering RNA (siRNA) silenced endogenous NFATc1 protein (Fig. 2A) and mRNA (Fig. 2B) in UMUC3 cells. Similarly, CsA and FK506 both at 1 μM reduced *NFATc1* gene expression in all bladder cancer cell lines tested (Fig. 2B). Subcellular localization of NFATc1 was then examined in UMUC3 by western blotting: treatment with CsA or FK506 resulted in decreases in nuclear NFATc1 expression as well as an increase in cytoplasmic NFATc1 expression (marginal change by CsA) (Fig. 2C). Inhibition of nuclear translocation of NFATc1 was further confirmed by immunofluorescence (Fig. 2D). Additionally, NFATc1-siRNA as well as CsA and FK506 decreased NFAT luciferase activity, compared with control-siRNA transfection or mock treatment (Fig. 2E). Significant but only 20% reduction in NFAT activity by the NFATc1-siRNA might be due to silencing of only one of NFAT isoforms. To confirm the down-regulation of NFAT activity by CsA and FK506, we measured expression levels of COX-2 and c-myc, downstream targets of NFATc1 signals [5, 8]. Significant decreases in COX-2 protein (Fig. 2F)/mRNA (Fig. 2G) and c-myc mRNA (Fig. 2H) by NFATc1-siRNA and CsA/FK506 were also seen. These results indicate that CsA and FK506 down-regulate the expression and activity of NFATc1 in bladder cancer cells.

### Anti-proliferative effects of CsA and FK506 in bladder cancer cells

**Figure 3:**
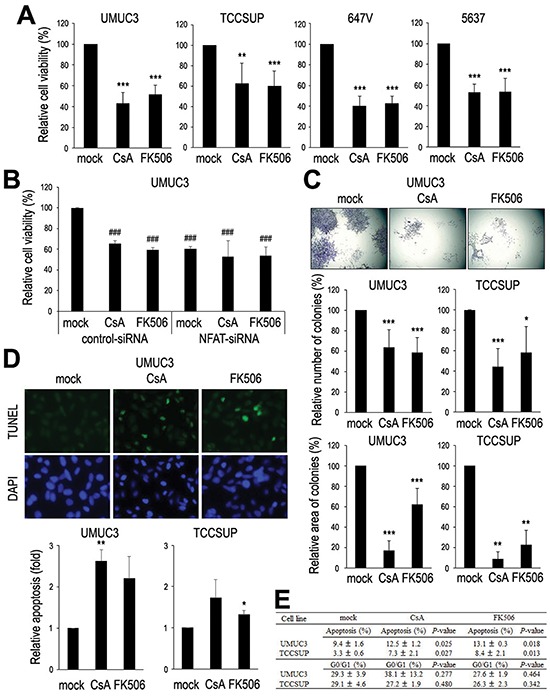
Effects of NFAT inactivation on bladder cancer cell proliferation MTT assay in UMUC3/TCCSUP/647V/5637 **(A)** or UMUC3 transfected with control-siRNA or NFATc1-siRNA **(B)**. The cells were cultured in the presence of ethanol (mock), CsA (1 μM), or FK506 (1 μM) for 4 days and their viability was assessed with MTT. Growth suppression is presented relative to that of mock treatment in each cell line (A) or mock treatment/control-siRNA expression (B). Each value represents the mean (+SD) from at least three independent experiments. **P < 0.01 (*vs*. mock treatment). ***P < 0.001 (*vs*. mock treatment in each cell line). ^###^*P* < 0.001 (*vs*. mock treatment in control-siRNA cells). **(C)** Clonogenic assay in UMUC3 and TCCSUP cells cultured in the presence of ethanol (mock), CsA (1 μM), or FK506 (1 μM) for 2 weeks. The number of colonies and their areas quantitated, using the ImageJ software, are presented relative to those of mock treatment in each cell line. Each value represents the mean (+SD) from at least three independent experiments. *P < 0.05 (*vs*. mock treatment). **P < 0.01 (*vs*. mock treatment). ***P < 0.001 (*vs*. mock treatment in each cell line). **(D)** TUNEL assay in UMUC3 and TCCSUP cells cultured in the presence of ethanol (mock), CsA (1 μM), or FK506 (1 μM) for 24 hours. Apoptosis (percentage of TUNEL-positive cells) is presented relative to that of mock treatment in each cell line. Each value represents the mean (+SD) from at least three independent experiments. *P < 0.05 (*vs*. mock treatment). **P < 0.01 (*vs*. mock treatment). **(E)** Flow cytometry in UMUC3 and TCCSUP cells cultured in the presence of ethanol (mock), CsA (1 μM), or FK506 (1 μM) for 24 hours. *P*-values (*vs*. mock treatment in each cell line).

To determine whether NFATc1 down-regulation exerts an influence on the proliferation of bladder cancer cells, we assessed cell viability [by methylthiazolyldiphenyl-tetrazolium (MTT) assay] and colony formation (by clonogenic assay) in bladder cancer lines treated with CsA or FK506. In the parental cell lines, CsA and FK506 strongly suppressed their growth (Fig. 3A). Similar inhibition by CsA and FK506 was observed in UMUC3-control-siRNA cells (Fig. 3B). However, there were no significant additional decreases in the growth of UMUC3-NFATc1-siRNA cells, while an inhibitory effect of NFATc1-siRNA, compared with UMUC3-control-siRNA, was seen (Fig. 3B), suggesting the suppression by CsA or FK506 predominantly via NFATc1. Both CsA and FK506 were also found to decrease the number and area of colonies in UMUC3 and TCCSUP cells (Fig. 3C).

To investigate how CsA and FK506 inhibit cell proliferation, we performed terminal deoxynucleotidyl transferase-mediated dUTP nick-end labeling (TUNEL) assay (Fig. 3D) and flow cytometry (Fig. 3E). CsA or FK506 treatment for 24 hours significantly increased apoptotic indices. By contrast, these only marginally changed the G0/G1 population.

### Suppressive effects of CsA and FK506 on bladder cancer cell migration and invasion

**Figure 4:**
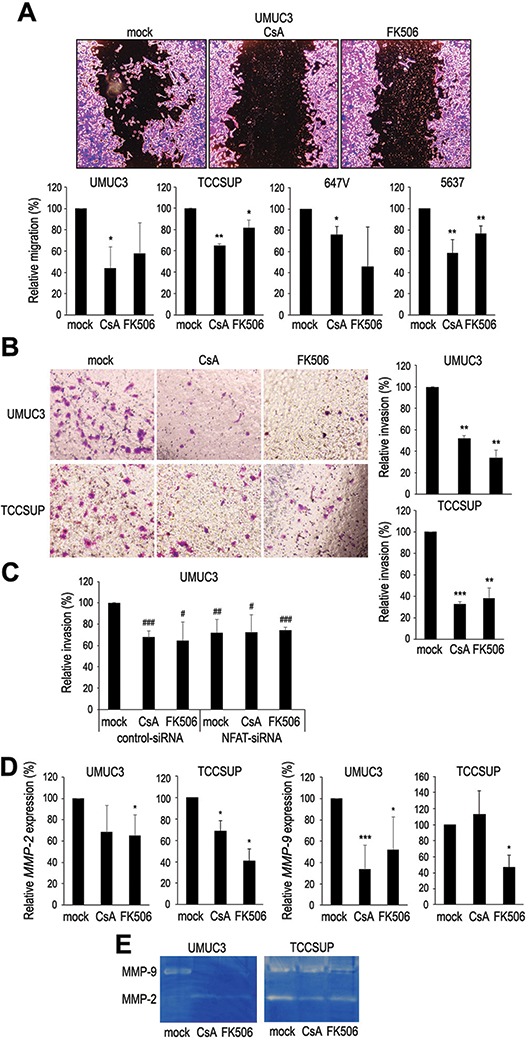
Effects of NFAT inactivation on cell migration and invasion **(A)** Wound healing assay in UMUC3/TCCSUP/647V/5637 cells. The cells grown to confluence were gently scratched and the wound area was measured after 24-hour culture with ethanol (mock), CsA (1 μM), or FK506 (1 μM). The migration determined by the rate of cells filling the wound area is presented relative to that of mock treatment in each cell line. Each value represents the mean (+SD) from at least three independent experiments. *P < 0.05 (*vs*. mock treatment in each cell line). **P < 0.01 (*vs*. mock treatment in each cell line). Transwell invasion assay in UMUC3/TCCSUP **(B)** or UMUC3 transfected with control-siRNA or NFATc1-siRNA **(C)**. The cells were cultured in the Matrigel-coated transwell chamber for 24 hours in the presence of ethanol (mock), CsA (1 μM), or FK506 (1 μM). The number of invaded cells present in the lower chamber was counted under a light microscope (10x objective in five random fields). Cell invasion is presented relative to that of mock treatment in each cell line (B) or mock treatment/control-siRNA expression (C). Each value represents the mean (+SD) from three independent experiments. **P < 0.01 (*vs*. mock treatment in each cell line). ***P < 0.01 (*vs*. mock treatment). ^#^*P* < 0.05 (*vs*. mock treatment in control-siRNA cells). ^##^*P* < 0.01 (*vs*. mock treatment in control-siRNA cells). ^###^*P* < 0.001 (*vs*. mock treatment in control-siRNA cells). **(D)** Quantitative RT-PCR of *MMP-2* and *MMP-9* in bladder cancer cells. UMUC3 and TCCSUP cells treated with ethanol (mock), CsA (1 μM), or FK506 (1 μM) for 24 hours were subjected to RNA extraction and subsequent real-time RT-PCR. Expression of each specific gene was normalized to that of *GAPDH*. Transcription amount is presented relative to that of mock treatment in each cell line. Each value represents the mean (+SD) from at least three independent experiments. *P < 0.05 (*vs*. mock treatment in each cell line). ***P < 0.001 (*vs*. mock treatment). **(E)** MMP-2/MMP-9 activity in bladder cancer cells. UMUC3 and TCCSUP cells cultured in serum-free medium in the presence of ethanol (mock), CsA (1 μM), or FK506 (1 μM) were subjected to gelatin zymography. The activity of MMP-2 (72 kDa) or MMP-9 (92 kDa) was indicated by clear zones of gelatin lysis against a blue background of stained substrate.

Cell migration and invasion are critical steps during tumor progression and metastasis. We therefore performed a scratch wound healing assay and a transwell invasion assay to assess the effects of NFATc1 inhibition on cell migration and invasion, respectively, in bladder cancer lines. In the wound healing assay, CsA and FK506 significantly delayed wound closure (Fig. 4A). Similarly, in the transwell assay, CsA and FK506 treatment demonstrated marked decreases in cell invasion ability (Fig. 4B). NFATc1 knock-down resulted in a significant decrease in the invasive properties, compared with the control line, while no significant effects of CsA and FK506 on cell invasion were seen in the NFATc1-siRNA line (Fig. 4C). Using a quantitative RT-PCR method, we then analyzed the effects of CsA and FK506 on the expression of matrix metalloproteinases (MMPs) that are known to play a critical role in cancer cell migration/invasion, angiogenesis, and resultant tumor progression and metastasis. CsA and FK506 decreased the levels of *MMP-2* and *MMP-9* expression, compared with the vehicle control, in two cell lines, except CsA for *MMP-9* in TCCSUP (Fig. 4D). We also determined the enzymatic activity of MMP-2 and MMP-9 by gelatin zymography and showed considerable decreases in those of MMP-9 in UMUC3 and MMP-2 in TCCSUP in the presence of CsA and FK506 (Fig. 4E).

### Anti-tumor activity of CsA and FK506 in a mouse xenograft model for bladder cancer

**Figure 5:**
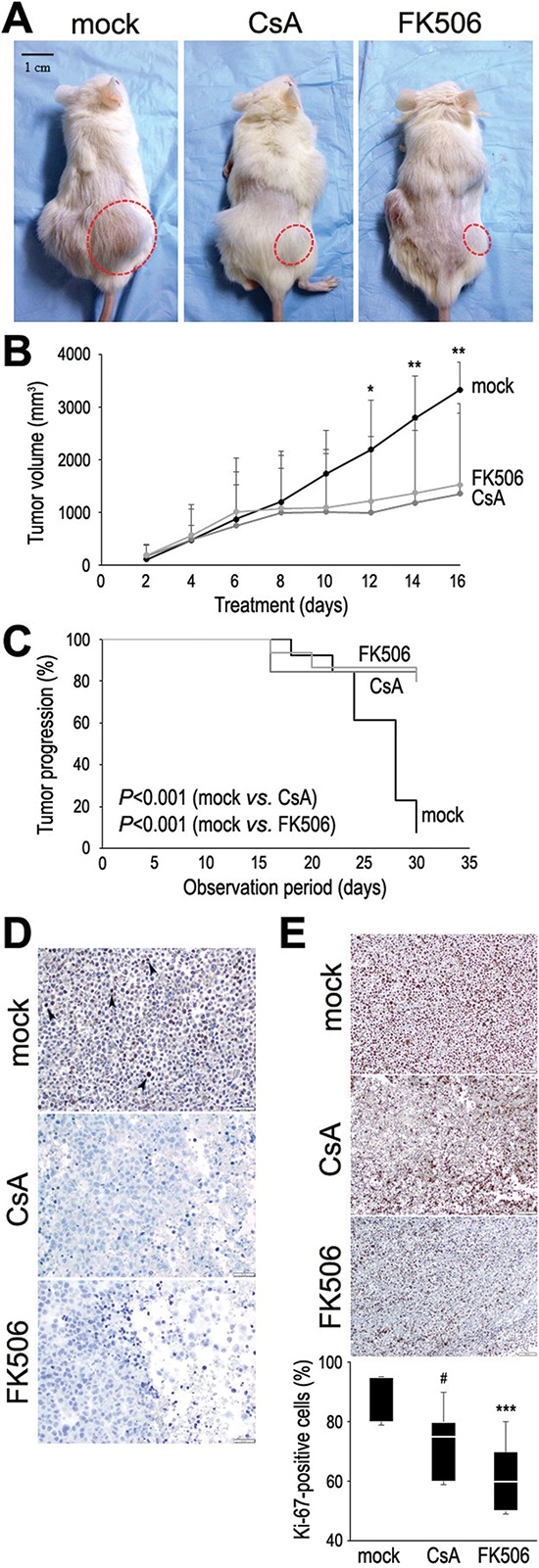
Effects of CsA and FK506 on tumor growth in a mouse xenograft model for bladder cancer **(A)** CsA (30 mg/kg/day), FK506 (3 mg/kg/day), or vehicle control was injected intraperitonealy in UMUC3-bearing NOD-SICD mice. **(B)** Tumor size was monitored every other days. *P < 0.05 (mock *vs*. FK506)/0.01 (mock *vs*. CsA). **P < 0.01 (mock *vs*. CsA or FK506). **(C)** Kaplan-Meier curves and log-rank test according to the endpoint set as tumor volume exceeding 2000 mm^3^ or 10% of animal's body weight. **(D)** Immunohistochemical staining of NFATc1 in harvested xenograft tumors. Occasional NFATc1 signals (arrowheads) are seen in tumors from control mice, but not in those from CsA/FK506-treated mice. Tumor necrosis is noted in CsA/FK506-treated tumors. **(E)** Immunohistochemical staining of Ki-67 in harvested xenograft tumors. Mean values (± SD) of the percentage of Ki-67-positive tumor cells are shown. ^#^Mock *vs*. CsA, *P* = 0.051. ***Mock *vs*. FK506, *P* < 0.001.

Finally, we used a mouse xenograft model to investigate whether CsA and FK506 inhibit bladder tumor growth *in vivo*. UMUC3 cells were implanted subcutaneously into the flank of SCID mice, and after 2–3 weeks, when the estimated tumor volume reached 100 mm^3^, we commenced daily injections of CsA or FK506 (Fig. 5A). As shown in Fig. 5B, the inoculated tumors in mice treated with CsA or FK506 were significantly smaller than those in the control mice at 12–16 days of treatment. CsA and FK506 also prevented the growth of tumors exceeding 2,000 mm^3^ or 10% of the animal's body weight (Fig. 5C). Immunohistochemical staining in the harvested specimens revealed decreases in NFATc1 expression (Fig. 5D) and cell proliferation as the percentage of Ki-67-positive cells (Fig. 5E) in CsA/FK506-treated tumors. These *in vivo* data further suggest that CsA and FK506 strongly inhibit the progression of bladder cancer.

## DISCUSSION

In contrast to a wide range of biological functions of NFATs in the immune system that have been recognized, an increasing, yet limited, amount of evidence suggests their involvement in the progression of solid tumors. In particular, their roles in bladder cancer progression remain largely unknown. In the present study, we demonstrate that NFATc1 inactivation via NFATc1-siRNA as well as CsA and FK506 results in bladder tumor regression.

Intracellular calcium-mediated calcineurin signaling and subsequent activation of the NFAT pathway, without specifying the involvement of its isoforms, have been implicated in cell proliferation of prostatic [13, 14] and endometrial [15] carcinomas. It has also been shown that NFATc1 activation promotes liver cancer cell proliferation [7] and breast cancer cell migration [9]. Potential downstream targets of NFATc1 for its regulation of cancer cell growth include c-myc, COX-2, and autotaxin [5, 7–9], all of which are known to involve stimulation of cancer cell proliferation and invasion, chemotaxis, and/or metastasis. Using bladder cancer lines, we here demonstrated that they overexpressed NFATc1 and that silencing of NFATc1 resulted in significant decreases, similar to those by CsA or FK506 treatment, in cell viability and invasion. These results further suggest that, among NFAT isoforms, NFATc1 plays a dominant role in bladder cancer progression. However, in other types of malignancies, other isoforms (*e.g*. NFATc2) were also shown to contribute to the promotion of tumor progression [16, 17], while inhibitory functions of NFATc2 [10] and NFATc4 [12] were noted. Therefore, each isoform is likely to specifically regulate downstream targets as well as tumor progression or regression in different types of cancers.

Correspondingly, overexpression of NFAT isoforms in several types of malignancies, other than bladder cancer, has been reported. Of these, the expression levels of NFATc1 were often elevated [5–7]. In these studies, localization of NFATc1 detected by immunohistochemical staining (*i.e*. nuclear *vs*. cytoplasmic signals) appeared to be cancer type-dependent. For instance, both nuclear and cytoplasmic signals were detected in pancreatic [5] and lung [6] carcinomas, whereas predominant nuclear/cytoplasmic expression was seen in hepatocellular carcinoma [7]/subcutaneous T-cell lymphoma [18], respectively. Our immunohistochemistry for NFATc1 in muscle-invasive bladder cancers stained both the nuclei and cytoplasms. Although there were no significant correlations between the status of NFATc1 expression and clinicopathologic features available for our patient cohort, those with nuclear NFATc1-positive tumor were found to have a significantly higher risk of disease progression. Our recent immunohistochemical study in separate sets of bladder TMAs consisting of non-muscle-invasive tumors [19] also detected both nuclear and cytoplasmic NFATc1 signals and further revealed significant increases in the level of its nuclear expression in urothelial neoplasm, compared with non-neoplastic urothelium, and in low-grade or high-grade urothelial carcinoma, compared with papillary urothelial neoplasm of low malignant potential, a very low grade tumor that is neither benign nor intrinsically malignant [20]. Our staining results thus support other data suggesting that NFATc1 promotes bladder cancer progression.

The immunosuppressive drugs CsA and FK506 have been shown to inhibit the phosphatase activity of calcineurin in T-cells and thereby prevent nuclear translocation of NFAT/inactivate the NFAT pathway [4, 21]. We confirmed this in bladder cancer cells by demonstrating that CsA and FK506 reduced the overall and nuclear levels of NFATc1 expression, suppressed the transcriptional activity of NFAT, and down-regulated the expression of COX-2 and c-myc. In addition, CsA and FK506 treatment inhibited cell viability, migration, and invasion of bladder cancer *in vitro*, and tumor growth *in vivo*. They also induced apoptosis, but not cell cycle arrest. Interestingly, these effects of CsA and FK506 were not seen in NFATc1 knock-down cell lines, suggesting that these non-selective NFAT inhibitors suppress bladder cancer growth predominantly through the NFATc1 pathway. Nonetheless, anti-proliferative effects of CsA via non-NFAT pathway have also been reported in colon cancer models [22].

As aforementioned, CsA and FK506 have often been prescribed in organ transplant recipients to prevent rejection as well as in a subset of patients with disorders related to autoimmunity. Treatment with these immunosuppressants is associated with potentially serious side effects that are often closely related to their blood concentrations [23]. As a result, the doses of CsA and FK506 are often strictly adjusted to maintain their levels of not more than 1200 ng/mL (1.0 μM) and 20 ng/mL (25 nM), respectively. Thus, FK506 clinically shows similar immunosuppressive effects with up to 50-fold lower doses than CsA [24]. In our cell line studies, the same concentration (1 μM) of CsA and FK506 achieved similar inhibition, whereas, in our xenograft model, 30 mg/Kg/day CsA and 3 mg/Kg/day FK506 showed similar effects. Therefore, CsA may be more potent in suppressing bladder cancer growth than FK506 at their pharmacological concentrations. Indeed, a pharmacological dose (25 nM) of FK506 did not significantly inhibit cell viability of bladder cancer lines (data not shown). In prostate cancer lines, CsA showed more significant inhibitory effects on cell proliferation, compared with FK506 at the same concentrations up to 10 μM [25]. Moreover, CsA was shown to inhibit the growth of both androgen-sensitive and androgen receptor (AR)-negative prostate cancer lines, while FK506 inhibited only AR-dependent growth but failed to do in AR-negative cells [25]. Although some bladder cancer lines are known to possess a functional AR [26–28], FK506 (and CsA) similarly inhibited the growth of AR-positive (*i.e*. UMUC3, TCCSUP) versus AR-negative (*i.e*. 647V, 5637) bladder cancer cells. Additionally, to prevent severe adverse drug reactions potentially seen with CsA or FK506 treatment, a peptide that interferes with the calcineurin-NFAT interaction [29, 30] and small molecule inhibitors of NFATs [31–33] have been developed and assessed mainly in animal models. The efficacy of these inhibitors in bladder cancer growth may also need to be tested.

In conclusion, NFATc1 likely plays an important role in bladder cancer progression. Our findings may also offer a potential therapeutic strategy for bladder cancer via targeting the calcineurin-NFAT pathways, especially NFATc1 signals. CsA (and FK506) may thus be able to be applied to the treatment of advanced bladder cancer. Further assessment of the functions of NFATc1 as well as other isoforms is required to determine biological significance of NFAT signaling in bladder cancer.

## MATERIALS AND METHODS

### Cell culture and chemicals

A human normal urothelial cell line (SVHUC) and human urothelial carcinoma cell lines (UMUC3, TCCSUP, and 5637) were originally obtained from the American Type Culture Collection. 647V cell line was used in our previous study [26]. All these lines were recently authenticated, using GenePrint 10 System (Promega), by the institutional core facility. SVHUC cells were maintained in F-12K (Mediatech) and other cell lines were maintained in Dulbecco's modified Eagle's medium (DMEM; Mediatech), all supplemented with 10% fetal bovine serum (FBS), penicillin (100 units/mL), and streptomycin (100 units/mL) at 37°C in a humidified atmosphere of 5% CO_2_. Cells were cultured in phenol red-free medium supplemented with 5% charcoal-stripped FBS (CS-FBS) at least 24 hours before experimental treatment. A siRNA targeting NFATc1 (sc-29412; Santa Cruz Biotechnology) or a non-silencing control siRNA (sc-37007; Santa Cruz Biotechnology) was transfected at a final concentration of 20-80 nM into the bladder cancer lines, using GeneJuice (Novagen). We obtained CsA and FK506 from Abcam.

### Cell proliferation

We used MTT assay to assess cell viability, as described previously [34]. Briefly, cells (1–3 × 10^3^) seeded in 96-well tissue culture plates were incubated with DMEM supplemented with CS-FBS in the presence or absence of CsA or FK506. The media were refreshed every 48 hours. After 96 hours of treatment, 10 μL MTT stock solution (5 mg/mL; Sigma) was added to each well with 100 μL of medium for 4 hours at 37°C. The medium was replaced with 100 μL dimethyl sulfoxide, followed by incubation for 5 minutes at room temperature. The absorbance was then measured at a wavelength of 570 nm with background subtraction at 655 nm using luminometer (FLUOstar Omega, BMG Labtech).

### Reporter gene assay

Cells at a density of 50-70% confluence in 24-well plates were co-transfected with 250 ng of pGL4.30 NFAT reporter plasmid DNA (Promega) and 2.5 ng of pRL-TK plasmid DNA, using GeneJuice. After 18 hours of transfection, the cells were cultured in the presence or absence of CsA or FK506 for 24 hours. Cell lysates were then assayed for luciferase activity determined using a Dual-Luciferase Reporter Assay kit (Promega) and luminometer.

### RT and real-time PCR

Total RNA (0.5 μg) isolated from cultured cells using TRIzol (Invitrogen) was reverse transcribed using 1 μM oligo (dT) primers and 4 units of Omniscript reverse transcriptase (Qiagen) in a total volume of 20 μL. Real-time PCR was then performed, using SYBR GreenER qPCR superMix for iCycler (Invitrogen), as described previously [32]. The following primer pairs were used for RT-PCR: *NFATc1* (forward, 5′-GTCCCACCACCGAGCCCACTACG-3′; reverse, 5′-GACCATCTTCTTCCCGCCCACGAC-3′), *MMP-2* (forward, 5′-TACAGGATCATTGGCTACACACC-3′; reverse, 5′-GGTCACATCGCTCCAGACT-3′), *MMP-9* (forward, 5′-TGTACCGCTATGGTTACACTCG-3′; reverse, 5′-GGCAGGGACAGTTGCTTCT-3′), *COX-2* (forward, 5′-CTGGCGCTCAGCCATACAG -3′; reverse, 5′-CGCACTTATACTGGTCAAATCCC-3′), and c-myc (forward, 5′-ACCAGATCCCGGAGTTGGAA-3′; reverse, 5′-CGTCGTTTCCGCAACAAGTC-3′). *GAPDH* (forward, 5′-CTCCTCCACCTTTGACGCTG-3′; reverse, 5′-CATACCAGGAAATGAGCTTGACAA-3′) was used as an internal control.

### Western blotting

Protein extraction and western blotting were performed, as described previously [28, 34, 35] with minor modifications. We also used a nuclear and cytoplasmic extraction reagent kit (NE-PER, ThermoScientific) for obtaining separate nuclear and cytoplasmic fractions. Equal amounts of protein (30–50 μg) obtained from cell extracts were separated in 10% sodium dodecyl sulfate (SDS)-polyacrylamide gel electrophoresis (PAGE) and transferred to polyvinylidene difluoride membrane (Immun-Blot PVDF Membrane, Bio-Rad) by electroblotting. Specific antibody binding was detected, using an anti-NFATc1 antibody (clone 7A6; dilution 1:500; Abcam), an anti-COX-2 (dilution 1:2000; Cayman Chemical), or an anti-GAPDH antibody (clone 6C5; dilution 1:5000; Santa Cruz Biotechnology), and a secondary antibody (mouse IRDye 680LT or rabbit IRDye 800CW, LI-COR), followed by scanning with an infrared imaging system (Odyssey, LI-COR).

### Colony formation

Cells (1 × 10^3^) seeded in 6-well plates and cultured in the presence or absence of CsA or FK506 were allowed to grow until colonies in the control well were easily distinguishable. The cells were then fixed with methanol and stained with 0.1% crystal violet in phosphate-buffered saline (PBS). The number of colonies and their areas were quantitated using ImageJ software (National Institute of Health).

### Cell migration

In order to evaluate the ability of cell migration, a scratch wound healing assay was performed. Cells at a density of 90–100% confluence in 6-well plates were scratched manually with a sterile 200 μl plastic pipette tip, cultured in the presence or absence of CsA or FK506 for 24 hours, fixed with methanol, and stained with 0.1% crystal violet in PBS. The width of the wound area was quantitated, using ImageJ.

### Cell invasion

Cell invasiveness was determined, using a Matrigel (60 μg; BD Biosciences)-coated transwell chamber (8.0 μm pore size polycarbonate filter with 6.5 mm diameter; Corning), as described previously [34]. Briefly, cells (5 × 10^4^) in 100 μL of serum-free medium were added to the upper chamber of the transwell, whereas 600 μL of medium containing 5% FBS was added to the lower chamber. The media in both chambers contained ethanol, CsA, or FK506. After incubation for 24 hours at 37°C in a CO_2_ incubator, invaded cells were fixed, stained with 0.1% crystal violet, and counted under a light microscope.

### Gelatin zymography

The gelatinolytic activity of MMPs was determined by SDS-PAGE gelatin zymography. Cells at a density of 60–70% confluence in 10-cm dish were cultured in serum-free medium containing ethanol, CsA, or FK506 for 24 hours at 37°C in a CO_2_ incubator. The conditioned medium was concentrated using Amicon Ultra-4 centrifugal filter units (30 kDa, Millipore) according to the manufacturer's instructions. An equal volume of the zymogram sample buffer (Bio-Rad) was added to the concentrated medium followed by 10% SDS-PAGE in a gel containing 0.1% gelatin (Sigma). After electrophoresis, the gel was rinsed with a renaturing buffer (Bio-Rad) for 60 minutes to remove SDS, incubated overnight in a developing buffer (Bio-Rad) at 37°C with shaking, and stained with 0.25% Coomassie Brilliant Blue R-250 (Bio-Rad). MMP activities were detected as non-staining regions in the gel, using ChemiDoc XRS+ System (Bio-Rad).

### Immunofluorescent staining

Cells plated onto 8-well chamber slides (NuncLab-Tek, Thermo Scientific) were cultured in DMEM with 5% CS-FBS containing ethanol, CsA, or FK506 for 24 hours. At the end of the drug treatment, the adherent cells were rinse twice with PBS and then fixed by 4% paraformaldehyde. The cells were blocked with 1% bovine serum albumin for 1 hour at 37°C, and a primary antibody (NFATc1; clone 7A6; dilution 1:50; Santa Cruz Biotechnology) was added and incubated overnight at 4°C. 4′,6′-diamidino-2-phenylindole (DAPI) was used to visualize nuclei. Fluorescence images were acquired with a fluorescence microscopy (EVOS FL Auto, Life Technologies).

### Apoptosis and cell cycle analysis

The TUNEL assay was performed on cell-burdening coverslips, using the DeadEnd Fluorometric TUNEL system (Promega), followed by counterstaining for DNA with DAPI. Apoptotic index was determined in the cells visualized by the fluorescence microscopy (EVOS FL Auto). For cell cycle analysis, flow cytometry was performed. Cells (1 × 10^6^/10-cm dish) were cultured in medium supplemented with CS-FBS containing ethanol, CsA, or FK506 for 24 hours, harvested with trypsin, fixed in 70% ethanol, and stained with propidium iodide (PI) buffer (50 μg/mL) for 60 minutes. Cellular PI content was measured on a Guava PCA-96 Base System^TM^ flow cytometer (EMD Millipore) equipped with a green laser at 532 nm wavelength. Data were analyzed, using the Guava Cell Cycle software (EMD Millipore).

### Mouse xenograft model

Animal protocols in accordance with the National Institute of Health Guidelines for the Care and Use of Experimental Animals were approved at our institution. UMUC3 (1 × 10^6^ cells/100 μL/site) resuspended in Matrigel (BD Biosciences) were subcutaneously injected into the flank of 6-week-old male immunocompromised NOD-SCID mice (Johns Hopkins Animal Resources). Treatment was initiated when the tumor volume reached 100 mm^3^. Mice intraperitoneally received 30 mg/kg/day CsA, 3 mg/kg/day FK506, or vehicle control (corn oil) once-daily. Serial caliper measurements of perpendicular diameters were used to calculate tumor volume by the following formula: (short diameter)^2^ × (longest diameter) × 0.5.

### Bladder TMA and immunohistochemistry

We retrieved bladder tissue specimens obtained by transurethral resection or cystectomy performed at the Johns Hopkins Hospital and University of Rochester Medical Center. Appropriate approval from the institutional review board at each institution was obtained before construction and use of the TMA. Bladder TMAs, consisting of 65 cases of muscle-invasive high-grade urothelial carcinoma, were constructed from formalin fixed paraffin embedded specimens, as described previously [36]. All these patients, including 51 men and 14 women with a mean/median age of 64.5/66 years (range: 40–89 years) and a mean/median follow-up of 35.4/16 months (range: 2–268 months), ultimately underwent cystectomy. None of the patients had received therapy with radiation or anti-cancer drugs prior to the collection of the tissues included in the TMAs.

Immunohistochemical staining was performed on the sections (5 μm thick) from the bladder TMAs or mouse xenograft tumors, as described previously [34–36]. Briefly, after deparaffinization, hydration, and antigen retrieval, samples were incubated overnight at 4°C with a primary antibody to NFATc1 (clone 7A6; dilution 1:50; Santa Cruz Biotechnology) or Ki-67 (clone 30-9; prediluted; Ventana) and then with a broad spectrum secondary antibody (Invitrogen). All stains were manually quantified by a single pathologist (H.M.) blinded to sample identify. For NFATc1 staining in the bladder TMAs, the German immunoreactive scores calculated by multiplying the percentage of immunoreactive cells (0% = 0; 1–10% = 1; 11–50% = 2; 51–80% = 3; 81–100% = 4) by staining intensity (negative = 0; weak = 1; moderate = 2; strong = 3) were considered negative (0; 0–1), weakly positive (1+; 2–4), moderately positive (2+; 6–8), and strongly positive (3+; 9–12).

### Statistical analysis

The Fisher exact test and the χ^2^ test were used to evaluate the associations between categorized variables. The numerical data were compared by Student's *t*-test. Correlations between variables were determined by Spearman's correlation analysis. Survival rates in patients were calculated by the Kaplan-Meier method and comparison was made by log-rank test. *P* values less than 0.05 were considered to be statistically significant.
